# Volumetric Segmentation of Cell Cycle Markers in Confocal Images Using Machine Learning and Deep Learning

**DOI:** 10.3389/fpls.2020.01275

**Published:** 2020-08-28

**Authors:** Faraz Ahmad Khan, Ute Voß, Michael P. Pound, Andrew P. French

**Affiliations:** Schools of Computer Science and Biosciences, University of Nottingham, Nottingham, United Kingdom

**Keywords:** plant analysis procedures, machine learning, deep learning, phenotyping, software, annotation

## Abstract

Understanding plant growth processes is important for many aspects of biology and food security. Automating the observations of plant development—a process referred to as plant phenotyping—is increasingly important in the plant sciences, and is often a bottleneck. Automated tools are required to analyze the data in microscopy images depicting plant growth, either locating or counting regions of cellular features in images. In this paper, we present to the plant community an introduction to and exploration of two machine learning approaches to address the problem of marker localization in confocal microscopy. First, a comparative study is conducted on the classification accuracy of common conventional machine learning algorithms, as a means to highlight challenges with these methods. Second, a 3D (volumetric) deep learning approach is developed and presented, including consideration of appropriate loss functions and training data. A qualitative and quantitative analysis of all the results produced is performed. Evaluation of all approaches is performed on an unseen time-series sequence comprising several individual 3D volumes, capturing plant growth. The comparative analysis shows that the deep learning approach produces more accurate and robust results than traditional machine learning. To accompany the paper, we are releasing the 4D point annotation tool used to generate the annotations, in the form of a plugin for the popular ImageJ (FIJI) software. Network models and example datasets will also be available online.

## Introduction

Understanding plant growth is becoming increasingly important, as the ability to feed an increasing population is heavily dependent on successful and efficient crop production. Growth of plants occurs as an interaction between two key activities: cell division and cell expansion. In developmental biology, quantifying cell divisions is an important measure to estimate and compare organ or tissue growth between different genotypes or to compare different growth conditions. In plants, the root meristem is located at the root tip and overall root growth is achieved by the sum of generating new cells by cell division and their subsequent elongation. Therefore, quantifying cell division is important when determining root growth dynamics. Being able to analyze these events is critical in many plant science experiments. Modern microscopy methods such as confocal or light sheet microscopy allow a biologist to see inside a plant root, at a cellular scale, and even over time. Analysis of these datasets for morphological changes remains challenging, as they often comprise large, possibly 4D image data. Certain fluorescent markers can be used to help visualize events such as cell division, but nevertheless, finding and counting the markers in these expansive datasets remains a labor intensive task.

Three-dimensional confocal microscopy is an imaging technology that allows us to see inside biological samples. Fluorescent markers or dyes are excited by laser light, allowing for clear labeling of particular structures within an organism. Volumetric image datasets can therefore be produced allowing inspection at the cellular scale. If timelapse imaging is used, such volumes can be collected at regular time points. The resulting “4D” datasets are large, and due to their dimensionality are hard to inspect by hand. Labeling regions in 3D data is often reduced to labeling 2D slices or drawing complex surfaces. Both methods are time consuming and often frustrating to perform in practice. This challenge is the motivation behind the study performed here: to determine the success of segmenting and locating fluorescent nuclei markers inside an Arabidopsis root using machine (classical and deep) learning methods. The particular markers used in this paper provide a unique challenge, as the structures are small and sparse, often ill-defined, and are marked by the same colour as other distracting features in the image such as the cell walls. We believe this therefore provides a tough test, applicable to a wide variety of real-world markers and features in confocal images.

Segmentation of nuclei in 3D has presented image analysis with a challenge for many years. Indeed, labeling and segmenting cells and sub-cellular features such as nuclei has been an active research area for decades (Meijering, 2012). There is a need across many biological disciplines to locate, count, and segment nuclei and nuclear markers for quantification in many types of experiments (Xing and Yang, 2016). Until recently, handling the challenges of nuclei segmentation, especially when in close proximity, has traditionally been accomplished by concatenating a pipeline of various analysis techniques together to separate the features in the image [e.g., (Mathew et al., 2015; Xing and Yang, 2016)]. Now, machine learning-based methods are providing more reliable results across a wider range of datasets without the need to use many individual processing steps. Deep machine learning in particular has begun to challenge preconceptions about how to accomplish image analysis tasks. Here, we present two approaches to a classic problem in confocal microscopy: fluorescent marker segmentation and localization. In particular, we are segmenting cell-cycle nuclear markers in 4D datasets; although the approach is flexible in nature and could be adapted for detection of similar nuclear markers. To do this, first, we explore a number of classical machine learning approaches to find the markers, and evaluate their segmentation accuracy. Second, we then build and test a modern deep machine learning approach to segment cell-cycle nuclear markers in 4D datasets, thereby developing an AI-based solution to the problem, operating on full volumetric (ie., *x*, *y*, *z*) datasets. We then compare and contrast the methods, as well as using the deep learning approach in a final counting scenario, were we are interested in counting how many cells are flagged by the marker (rather than stopping at labeling pixels).

## Related Work

It is certain that over coming years, we will see a trend for traditional image analysis and processing pipelines to be replaced or at least supported by dedicated deep learning models. Some existing popular tools for cell image analysis, such as CellProfiler (McQuin et al., 2018), have recently begun to support the ability to load in developed deep learning models to form part of their analysis suite. Beyond segmentation, other applications of deep learning with reference to microscopy include approaches to generate super-resolution images—software-enhanced images creating clarity beyond the physical limits of the microscope system in use (Wang et al., 2019).

In recent years, traditional computer vision and machine learning has been used in a variety of fields to perform image segmentation. Image analysis at the textural level (i.e., considering properties of local pixel patches) has been used by many authors when wanting to produce a segmented output. Zayed and Elnemr (2015) proposed a method for detecting and segmenting abnormalities in Computed Tomography (CT) images of lungs, such as pulmonary edema and tumors. The authors relied on Haralick features based on Gray Level Co-occurrence Matrix (GLCM) to extract textural patterns from the lung images. Haralick features are a set of statistical measurements that effectively describe the overall texture of the image. Similarly, Chaddad et al. (2011) relied on five measurements of the Haralick features for classifying and segmenting colon cancer cells in multi-spectral bio-images. Fleet et al. (2014) used Haralick features to capture textural patterns from image reconstructions of ultrawideband microwave scans. They demonstrated the feasibility of Haralick features for breast cancer detection by classifying between malignant tumor present and no malignancy found. Recently, Brynolfsson et al. (2017) analyzed Haralick features extracted from apparent diffusion coefficient (ADC) MRI Images and assessed the sensitivity of these features against five image acquisition parameters. Similarly, Campbell et al. (2017) also utilized Haralick features in a recent study for textural analysis of brain positron emission tomography (PET) images. In addition to being effectively used in medical textural analysis, Haralick features have also been effectively used in the quality control field (Corbane et al., 2008; Bhandari and Deshpande, 2011). As a choice of feature, then, Haralick seem to show a capability to segment regions in challenging image data across a variety of datasets.

Three-dimensional segmentation using deep learning presents particular challenges. Most deep learning to date has focused on 2D data, requiring significantly fewer computational resources due to the size of the datasets in use. Care must be taken when developing fully volumetric approaches, as the limits of even modern computer systems are easily reached. The work here focuses on plant cells, but 3D microscopy segmentation using deep learning is an active area of research in many domains. Recent work has developed a deep network approach to analyzing neurites in brain EM images (Zeng et al., 2017). Convolutional neural networks have also been used to segment larval zebrafish intestines in light sheet images (Hay and Parthasarathy, 2018). This was found to be as accurate as human experts and outperformed other non-deep machine learning approaches in common use, such as random forest and support vector machines. MRI (Magnetic Resonance Imaging), the medical 3D imaging technique, has also received attention from the deep learning community, applications of which include brain scan analysis (Akkus et al., 2017), and diagnostics for knee imaging (Bien et al., 2018).

When segmenting regions of interest in 3D, several deep learning architectures may be used, but all have a common structure. Key to any form of semantic segmentation is to enable an efficient upscaling in the network so that a high-quality, pixel-wise map can be produced for the segmentation labels. Convolutions are used to reduce the initial image dimension down to a spatially reduced set of dense, high-level features. These are then upsampled back into pixel values at a scale normally equivalent to the input image. As spatial information is lost in the central part of the architecture, techniques have been developed to maintain spatial information throughout this process. These include skip layers in the Fully Convolutional Network [e.g., (Long et al., 2015)] or in the case of U-Net, many feature channels are additionally used in the upscaling path to preserve features (Ronneberger et al., 2015). This latter 2D approach is expanded to handle volumetric data with 3D U-Net (Çiçek et al., 2016). In particular, the authors recognize the challenge of annotating 3D data to provide as training instances, so propose a 2D annotation approach. The network is envisioned to both fill in sparse 2D annotations to produce a 3D dataset segmentation, and to generalize to new unannotated datasets. Both applications are relevant to this work, so a 3D encoder-decoder architecture based on a modified U-Net will be the base architecture used here.

The rest of this paper is organized as follows. In the following section, we describe the nature of the microscopy involved and the dataset details. This is followed by the methodology section where the machine learning systems utilized and the proposed deep learning architecture are outlined. The results achieved on the dataset by all systems are also compared here and discussed. We then present quantitative and image-based results of the developed deep learning approach on unseen data, and the results are then explored in the discussion. This includes an examination of the failure cases of the network, and proposed methods of handling such errors in the future.

## Methodology

### The Confocal Dataset

The confocal dataset used here is composed of five 3D time-lapse sequences depicting five growing Arabidopsis thaliana roots. The purpose behind the time series is to ensure we capture cell divisions, hence confocal microscopy time course datasets have been generated. Visualizing cell division events is challenging, as the divisions (seen as the emergence of new cell walls) happens relatively quickly compared to the data capture rate, so this process is likely to occur between image capture points. Therefore, identification of dividing cells is facilitated here by the use of specialist marker lines, in which fluorescent cell division-specific proteins allow the visualization of dividing cells. In particular, we made use of the Arabidopsis thaliana line CYCB1;1:: CYCB1;1-GFP (Boudolf et al., 2004) which marks nuclei of dividing cells. Imaging was carried out on a Leica SP8 Confocal microscope. The fluorescing markers can be seen in the captured image as bright spots in **Figures 2A, C**, for example.

**Figure 1:**
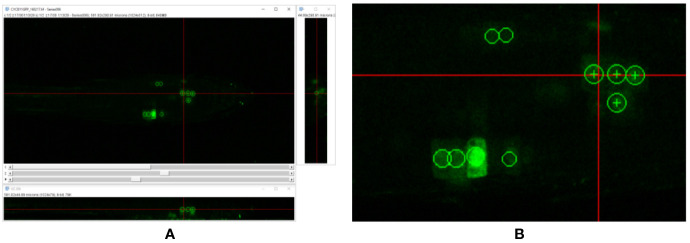
**(A)** Annotation of cell division events using the orthogonal pixels plugin in Fiji Schindelin et al., 2012. **(B)** Zoom of **(A)**. Note that point annotations are visualized as spheres—circles are seen in each of the orthogonal views. This makes labeling of 3D data in individual views easier for the annotator, and prevents common errors, such as double labeling of single structures. Crosses indicate the center of the sphere, indicating the plane on which they were placed.

To visualize and quantify this cell division in Arabidopsis, we imaged growing roots of 5-day-old seedlings (grown at 22°C in 12-h light 12-h dark cycles on 0.5 MS, pH 5.8) for up to 3 h, every 10–30 min. To train the software, every cell division event was manually annotated using “Orthogonal Pixels” in FIJI (Schindelin et al., 2012). This is a custom written plugin which alleviates the challenge of labeling broadly spherical 3D structures as points in volumetric data. The problem with annotating such data is that it can be a challenge to identify where the center of such a structure lies, and additionally in identifying if a structure has already been labeled on a neighboring slice. This tool allows efficient capture of point annotations in 3D or 4D by representing 3D clicks visually as spheres. At each stack, spheres representing clicks in nearby slices are also visualized, reducing the chance of annotating the same object twice (see **Figure 1**). This plugin is available as a supplemental file to accompany this paper (see Supplemental Data).

In total, a little over 1,000 cell division events were annotated. To enable efficient labeling, all such events in the dataset have been annotated by a single expert, stored as *x*, *y*, *z*, and *t* coordinates representing the center of each nucleus in 3D space and at a point in time. These have been manually localized in the datasets by navigating to the *x*, *y*, *z*, *t* position within the data using the plugin, and adding an annotation with the space bar. Annotations are then stored as a CSV text file alongside the volumetric image data. Note that although individual time points are considered here for the marker localization task, we anticipate the approach being deployed on data captured over longer periods and more frequent time points using technology such as light sheet microscopy. A comparison of confocal microscopy and lightsheet microscopy and the challenges of imaging with plants can be found in Ovecka et al. (2018). Importantly, the data resolves to ultimately the same format as used here, both being volumetric RGB image stacks, exported from the manufacturer’s microscopy software as uncompressed TIFF files.

The point *x*, *y*, *z* coordinates at a specific time need further processing in order to effectively train a semantic segmentation network. Our point labels must be expanded to encompass a volume of space, which is centered on the feature we are interested in. To this end, in order to generate a ground truth volume for training purposes, a 3D Gaussian function is used to generate a region of interest at every *x*, *y*, *z* coordinate. This provides a contextual volume of space to the network centered on the area of interest. As the marker is concentrated in the nucleus with some bleed into the surround cell space and surrounding space, this is an appropriate representation.

(1) $$G(x,y,z)=\frac{1}{2\sigma^{2}}\text{exp}(-({\left(x-x_{0}\right)}^{2}+{\left(y-y_{0}\right)}^{2}+{\left(z-z_{0}\right)}^{2})$$

**Figure 2:**
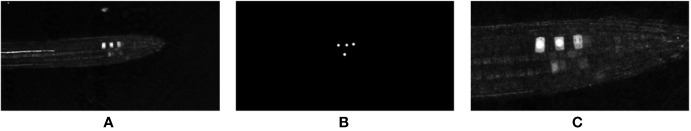
**(A)** Shows a 2D slice from a 3D confocal volume, and its corresponding ground truth can be seen in **(B)**. **(C)** shows zoomed details of panel **(A)**, highlighting the fluorescing cell-cycle markers, as well as bleed-through marker fluorescence and noise.

Here, *x*_0_, *y*_0_, *z*_0_ represent the center of the area of interest, and σ represents the standard deviation of the Gaussian curve. The standard deviation effectively defines an area of interest centered on the click locations. Depending on the loss function in use, this Gaussian can be used to define contextual interest which drops off away from the center, or a threshold can be applied to effectively produce fixed radius spheres around the annotation points. This latter approach produces a binary 3D volume where the areas of interest (nuclei in this case) are represented in a sphere defined by logical 1’s and anything other than the nuclei are represented by logical 0’s. A 2D slice from a 3D volume can be seen in **Figure 2A**, while its corresponding ground truth can be seen in **Figure 2B** where a 3D Gaussian is generated at every point of interest from a ground truth coordinate file accompanying the image data. In the case of **Figure 2B**, this is then thresholded at a fixed distance to produce a spherical region.

As explained above, the confocal dataset is composed of five time-lapse sequences comprising roots of five different plants, which contain in total 142 individual time points. Out of these five sequences, four (containing 136 time points total) are used for training (132) and validation (4) while the last (containing 6 time points) is used for evaluating final performance of the network. At no time during training is any volume from the test sequence introduced to the network, ensuring adequate separation between training and testing, and allowing a “real world” demonstration of the network as if run on a newly captured time sequence in a subsequent biological experiment. We used this approach rather than randomly assigning individual time points to test and train sets to avoid close correlations between testing and training, i.e., neighboring time points could be separated into training and testing, while being similar in appearance. However, we note this does leave limited data for testing, and means the test set is challenging as it is drawn from a separate capture session.

#### Classical Machine Learning

To begin with we will introduce and implement some classical machine learning approaches to the task; both supervised and unsupervised algorithms will be implemented. While we anticipate these approaches to have some success, we also anticipate challenges with the complex images present here, which we hope the subsequent deep learning approach will overcome. Supervised machine learning approaches use manual ground truth with labeled volumes, where the goal is to learn a function that, when given new unseen data, is able to predict the output label of that data. Unsupervised learning on the other hand does not require any ground truth and the goal is to infer the underlying distribution or structure of the given data. We provide here a brief consideration of some common methods as comparison; this is not intended as an exhaustive study of machine learning approaches.

#### Unsupervised Learning

To begin, we implement an unsupervised learning algorithm, k-means clustering (Lloyd, 1982). K-means aims to divide the data points into “K” clusters, a cluster being an aggregation of data points having certain similarities. For our purposes, clustering will be performed based on pixel intensities.

**Figure 3:**
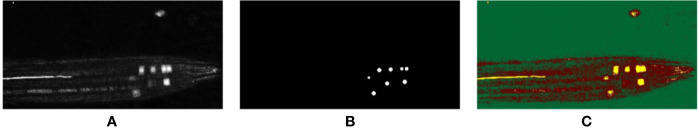
2D representation of the **(A)** input volume, **(B)** the accompanying ground truth, and the **(C)** segmentation results achieved via k-means clustering; green: background, red: non-nuclei foreground, yellow: nuclei.

From the input image shown in **Figure 2**, we can observe that the entire sample, based on intensities, can be grouped into three main regions: the background, the foreground and the bright nuclei. Based on this observation, clustering is performed on the test volume with three clusters (i.e., k = 3), with the intention of each region being represented by a cluster. K-means clustering is an iterative algorithm that continues until cluster assignments of the data points stop changing below a certain epsilon value (0.2 used here). Once all pixels are grouped into their respective clusters, we can separate our cluster of interest (representing the marker) from the others. This is achieved with the help of a masked image that only displays the pixels of the cluster related to the nuclei while ignoring the other two clusters. The pixel-distribution of a single test volume while using each cluster as a mask is given in **Table 1**. Looking at **Figure 3C**, cluster 2 appears to provide the best segmentation of the nuclei marker in our method, but an optimal k-means approach may require more complex handling, such as combining, of a larger number of clusters.

**Table 1:** Pixel distribution of each cluster using K-means clustering on a single test volume.

	Background (pixels)	Foreground (pixels)	Nuclei (pixels)
**Cluster 1**	9786380	1223668	4597
**Cluster 2**	10972171	37877	6021
**Cluster 3**	2627172	8382876	437

As can be seen, despite the simple implementation method requiring no training data, and being based on intensity values alone, the k-means algorithm was able to group all the pixels representing nuclei into a single cluster. But since this is based on an intensity measure alone (i.e., without spatial context), pixels having intensities similar to the nuclei are also grouped into the same cluster, which is evident from the output, where the location and shape of regions is not taken into account by the algorithm. For our simple scenario here, k-means appears to produce acceptable results without any supervised training, albeit including some irrelevant biological features into the segmentation, but for more complex images this method is likely to significantly under-perform. By providing training data via supervised learning, we hope to improve this result.

#### Supervised Learning

Supervised machine learning typically produces much more accurate results than unsupervised methods, as the algorithm is allowed to learn the relationship between input and the expected output. However, unlike unsupervised methods, supervised learning cannot work on the sample directly and needs to be trained using a dedicated training dataset. Furthermore, descriptive features must be extracted from the training dataset before they can used in order to produce a more effective predictive model. Raw pixels often do not contain enough information for a system to learn effectively, so a number of information-rich features must be extracted from the image data first. A range of suitable supervised machine learning algorithms and feature extraction methods have been analyzed here, with a view to comparing the results achieved here with the deep learning approach proposed later in this paper.

#### Feature Extraction

For any machine learning algorithm to be effective, a well thought out feature extraction and selection pipeline is necessary. As we are aiming to perform segmentation (i.e., per pixel classification), we will explore textural features, i.e., features that retain the original image dimensions, and by doing so maintain the per pixel connection with the ground truth volume. By being limited to textural features many of the otherwise-effective, well-known feature extractors such as Scale Invariant Feature Transform (SIFT) (Lowe, 2004), Speeded Up Robust Features (SURF) (Bay et al., 2008), Bag of Visual Words (BoV) (Csurka et al., 2004), Features from Accelerated Segment Test (FAST) (Rosten and Drummond, 2005), Oriented FAST, and Rotated BRIEF (ORB) (Rublee et al., 2011) would not be suitable in our case.

Textural feature extraction methods analyze the spatial distribution of gray values and compute local features at each pixel by inferring often statistical measures from the local distribution of features. Based on the literature review performed, it has been established that to perform segmentation of images textural features must be utilized and that Haralick features have been demonstrated to be a strong candidate for textural analysis of images. Taking guidance from previous studies and their findings, we have also utilized Haralick features for the textural analysis of our confocal dataset. Furthermore, in addition to Haralick features and for the sake of comparison we have also utilized the popular Local Binary Patterns (LBPs) which is another well-known and effective feature for textural analysis. To note, feature selection is important in machine learning as it is the foundation on which learning is built; it is also in contrast to deep learning, where features themselves are learnt and do not need to be selected. What we represent here are commonly used feature selection methods, rather than a necessarily optimal choice.

##### Haralick Textural Features

Haralick et al. proposed the Gray Level Co-occurrence Matrix (GLCM) and the set of textural measures extracted from the GLCM (Haralick et al., 1973). These features have since been widely used in biomedical image analysis. Haralick feature extraction consists of two steps, first the GLCM is determined by calculating the occurrence of a gray level at a certain geometric position for each pixel relative to its neighboring pixels. Next, a set of nine statistical measures are calculated from the GLCM that forms the textural representation of that image. The nine statistical measures are angular second moment, contrast, correlation, variance, sum variance, inverse difference moment, sum average, entropy, and sum entropy.

##### Local Binary Patterns (LBPs)

LBPs are textural descriptors that were made popular as a strong discriminatory feature by the work of Ojala et al. (Ojala et al., 2002) but were first proposed as early as 1993 in (Wang and He, 1990). LBPs determine the local representation of a texture by comparing each central pixel to its surrounding neighbors and in doing so, the image is encoded to a binary representation of its grayscale values. The neighbors smaller than the central pixel are set to 0 while those larger or equal to the central pixel are set to 1. A binary number is generated by concatenating the neighboring pixels in a clockwise manner and the resulting decimal value replaces the central pixel. LBP is represented mathematically as

(2) $$LBP_{N,R}(x,y)=\sum_{n=0}^{N-1}s(i_{n},i_{c})2^{N}$$

where *i_c_* represents the gray level value of the central pixel, *i_n_* represents the gray level values of *N* surrounding pixels in a neighborhood of radius *R*, and the functions *s*(*x*) is represented as

(3) $$s(x)=\{\begin{array}{cc} 1 & x\geq 0\\ 0 & x<0 \end{array}$$

#### Classifiers

Once features have been calculated from the raw image data, we then need algorithms to learn to classify data based on these features. The three machine learning classifiers implemented here (using the scikit-learn python library) are outlined below. We chose them as sensible and popular classifiers, rather than to claim they are optimal for this particular work.

##### Support Vector Machine (SVM)

Support Vector Machines, also known as maximum margin classifiers (Bishop, 2006), are discriminative classifiers that are formally defined using a separating hyperplane. A hyperplane is a decision boundary that classifies data points falling on either side of this boundary using their respective class. In two dimensions, this is simply a line. Support vectors are the data points that lie closest to the hyperplane and are the ones that are most difficult to classify, and as such have a direct bearing on the optimum location of the dividing hyperplane. SVM relies on the proper kernel selection and a regularization parameter to be an effective classifier, the selection of which has been described in *Implementation Details*.

##### Random Forests (RF)

Random Forests comprise an ensemble of randomized decision trees which use a bagging approach. A decision tree can be thought of as performing a series of “yes” or “no” decisions before eventually leading to a predicted class—a series of questions are asked before zeroing in on the predicted class label. A common problem with decision trees is that they tend to very easily overfit to the training data; a single tree can very easily fit to the details of that particular data rather than the overall distribution of the general properties of that dataset.

Random Forests on the other hand are a model that is composed of multiple decision trees, and demonstrates that multiple overfitting estimators when combined tend to reduce the overfitting effect. Random Forests average the final prediction of each tree in the forest and follow two key concepts in doing so: 1) When building trees, the training data is sampled randomly and 2) when splitting nodes, a random subset of features is considered.

##### Gradient Boosting Classifiers (GBC)

Gradient Boosting is also an ensemble learning method made from the combination of many decision trees, but differ from Random Forests in the way the trees are built, their order and how the results are combined. GBC builds one tree at a time, where every new tree learns from the errors of the previous tree and makes corrections accordingly (in contrast a Random Forest builds each tree independently). Since GBCs are built in a forward stage-wise manner all the results are combined along the way rather than averaging at the very end, as is done in a Random Forest model. GBCs are known to be an effective classifier when tuned properly, especially when dealing with highly imbalanced and sparse data (Frery et al., 2017), which makes them a hopeful candidate here.

#### Implementation Details

All results reported in this work have been produced on our local machine having an Intel Xeon 3.6 GHz processor and 16GB of RAM. The proposed deep learning architecture was trained using an NVIDIA GeForce GTX 1060 GPU.

As mentioned above, since a per-pixel classification is being performed each pixel effectively becomes a feature for training the classifiers. This produces an overwhelmingly large number of features (2.1 Billion pixels from the entire dataset) that would require an excessive amount of memory to process therefore, to avoid running out of memory during analysis and to avoid over-fitting the classifiers by using such a large number of features, 5,000 (out of 524,288) randomly selected features are retained from each image (slice) for the training process, while no random selection is performed for evaluation/testing, i.e., when testing or evaluating the network, all features of the test sample are used without any random selection of these features. Five thousand features per image is the maximum allowed number before running out of memory on our local machine; therefore, we found it better to select these features randomly rather than sequentially. This random selection of features provides a representation of the full training dataset, as the ratio of the nuclei to background pixels in the randomly selected features is found to be the same as in the full dataset (1:1,500); it allows features from the foreground to be selected despite being a very small class. This imbalance between foreground and background can be seen in, e.g., **Figure 2**, and training any classifier with such an imbalance would create a natural bias toward the majority class. This imbalance is tackled by performing a weighted training of the classifiers and is accomplished by assigning a higher misclassification penalty to the minority class (foreground in this case) during training.

#### Parameter Hyper-Tuning

Classifiers are parameterized so that their behavior can be tuned to the dataset on which they are trained and it is advised to find the best combination of these parameters using a validation sub-set before the training process can begin. Hyper-parameter tuning was performed on all classifiers by using a random search approach of 100 iterations and a three-fold cross-validation. The best result for the SVM model was achieved by using a regularization parameter, C, of 0.7 and a radial basis function (RBF) kernel. Similarly, the best fit for the RF classifier was achieved using 35 trees in the forest having a max depth of 17, 9 were the minimum samples required to split a node and 24 were the minimum samples required to be at a leaf node. The quality of the split was measured by the *gini* criterion. GBC produced the best results using 100 trees in the forest at a max depth of 1, the minimum samples per leaf and the minimum samples required for a split were found to be 0.2 and 0.3, respectively. The quality of each split was measured using the *Friedman mse* criterion.

### Results

**Figure 4:**
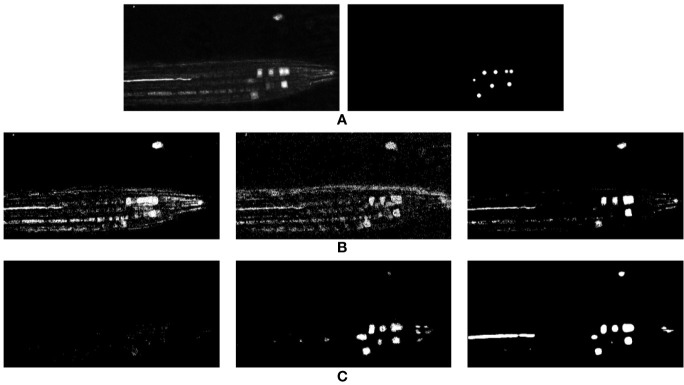
2D representation of the classification results achieved for a single test volume using machine learning. **(A)** Input volume and accompanying ground truth. **(B)** SVM, RF, and GBC predicted outputs using LBP. **(C)** SVM, RF, and GBC predicted outputs using Haralick features.

All machine learning operations are performed at the 2D level on each individual 2D slice, the results of which are concatenated at the end to get a 3D volumetric output. The middle output slice of a test volume is shown in **Figure 4** in an effort to visualize the output volume.

Numerical evaluation of each result performance is carried out by determining the F-measure value, which is defined as the harmonic mean of precision and recall, and also referred to as the F1 Score. Precision represents the ability of the network not to classify a sample as positive when it is actually negative, and is determined using

(4) $$Precision,p=\frac{tp}{\left(tp+fp\right)}$$

Conversely, the recall value is the ability of the network to detect all of the positive samples present in the volume and is determined using

(5) $$Recall,r=\frac{tp}{\left(tp+fn\right)}$$

where, *tp* is the number of true positives, *fp* is the number of false positives and *fn* is the number of false negatives. The F-measure is a combination of these values, giving an overall measure of performance and is determined as

(6) $$F-measure=2\left[\frac{\left(Precision)(Recall\right)}{\left(Precision+Recall\right)}\right]$$

The precision, recall and f-measure scores for all three classifiers have been reported in **Table 2** and visual results can be seen in **Figure 4**. **Figure 4B** represents the outputs predicted by SVM, RF, and GBC respectively for the input slice shown in (A) using LBP features, while **Figure 4C** represents the same but for Haralick features.

**Table 2:** Evaluation of supervised machine learning results using precision, recall, and f-measure, per-pixel measures using Haralick features over six time points in the test time-series.

Volumetric Time Stamp	Support Vector Machine (SVM)			Random Forest (RF)			Gradient Boosting Classifier (GBC)		
	Precision	Recall	F-Measure	Precision	Recall	F-Measure	Precision	Recall	F-Measure
1	0.0003	0.063	0.0006	0.190	0.438	0.258	0.077	0.493	0.132
2	0.002	0.185	0.004	0.455	0.464	0.339	0.115	0.557	0.179
3	0.0007	0.073	0.001	0.436	0.519	0.432	0.126	0.756	0.212
4	0.0002	0.038	0.0005	0.419	0.544	0.431	0.099	0.843	0.174
5	0.0001	0.042	0.0003	0.135	0.365	0.185	0.071	0.526	0.118
6	0.000	0.0022	0.000	0.081	0.289	0.116	0.053	0.521	0.092

Out of the three classifiers, SVM produced the worst results using either feature. This is not unexpected as SVM classifiers are known to be effective where the dimensionality of a feature is much larger than the number of features. Our case here is the opposite, as we have a very large number of features with each having a very small number of variables per feature. On the other hand, the decision tree classifiers produced comparatively better results, and it can be seen that out of the two ensemble methods used, GBC produced a better result for such an imbalanced and sparse datase, possibly due to the forward stage-wise construction of the GBC trees. GBC trained on Haralick features produced the cleanest results with minimum pixel noise in **Figure 4**, but was still unable to differentiate between the nuclei and any artefacts in the image as can be seen from the last panel of **Figure 4C**. Despite a noisier visual output, Random Forests had a slightly improved F-Measure (**Table 2**) compared to GBC, but both methods far outperformed the SVM method. Furthermore, although Haralick features produce the best results (**Figure 4B** versus **4B**), they have a very long inference time (we experienced an inference time of approximately 12 h per input volume). This is due to the fact that each 3D volume is broken down into patches of size [11 × 11] from which haralick features are calculated. Having such a long inference time, this may not be a practical solution.

### Deep Learning

**Figure 5:**
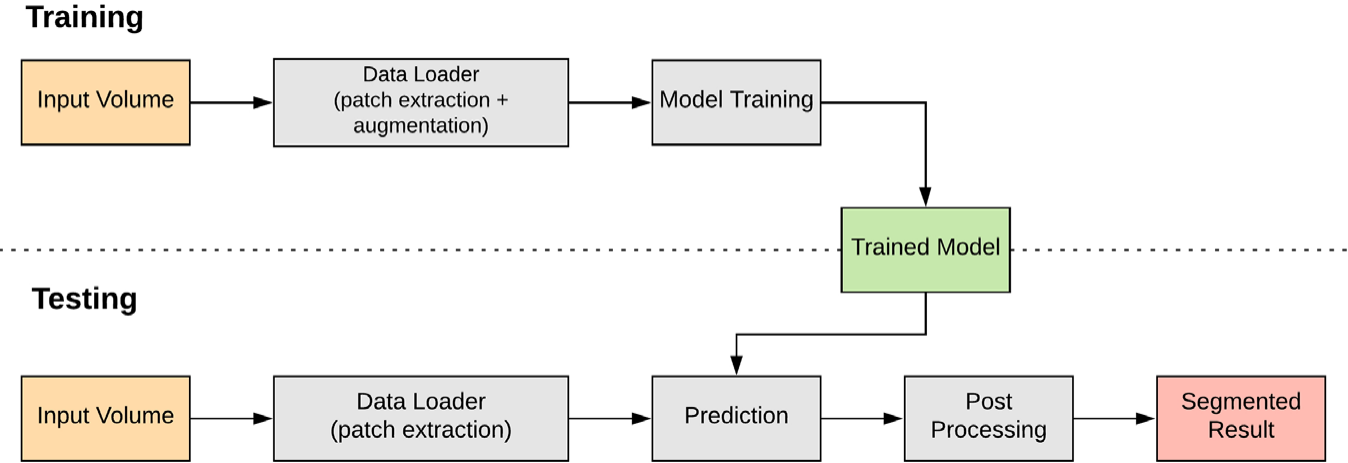
A pipeline view of our proposed deep learning approach.

This section discusses the deep learning approach to classifying and locating the nuclei markers in our dataset. A pipeline of the deep-learning approach is shown in **Figure 5**. One key advantage with deep learning is we no longer have to choose our own features; deep learning will learn appropriate features as part of the model.

**Figure 6:**
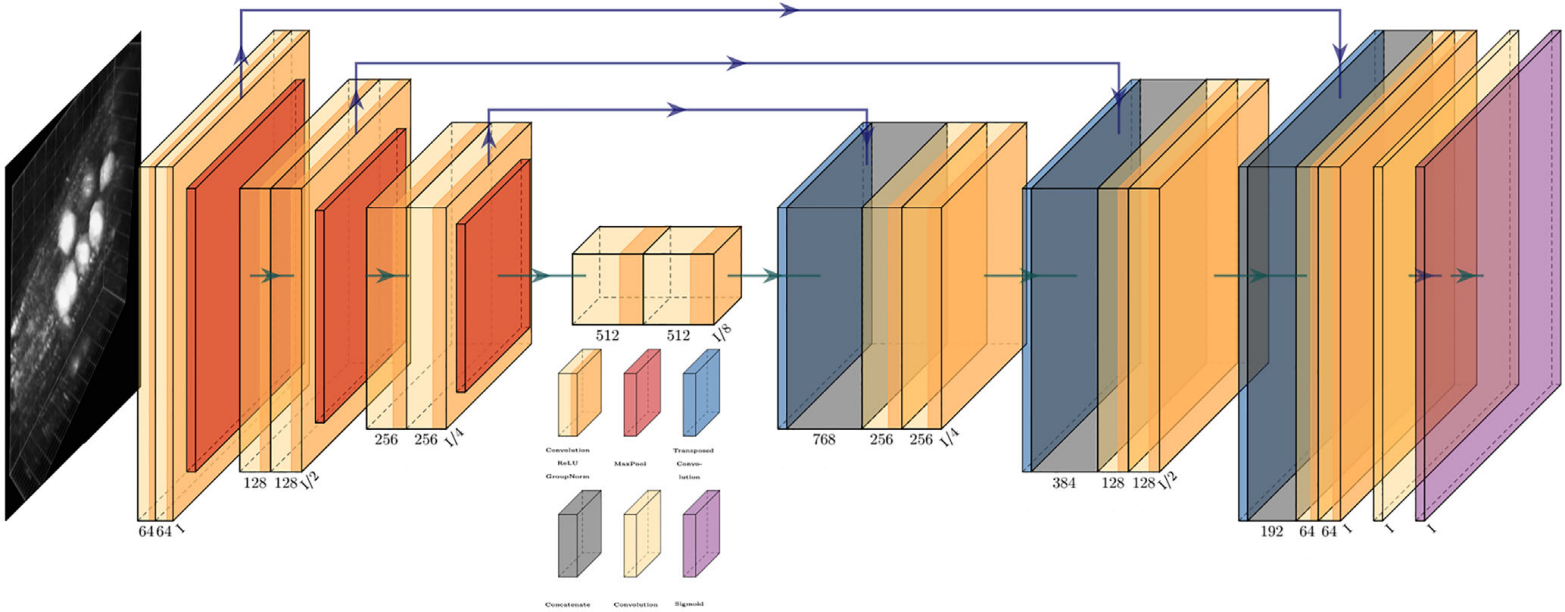
An overview of the network architecture used. Key to blocks: yellow, convolution; red, max pooling; blue, transposed convolution; gray, concatenation; purple, sigmoid.

The network proposed here utilizes the encoder-decoder approach based on a modified version of the 3D U-Net presented in (Çiçek et al., 2016). Such deep learning networks have many design parameters such as the number and depth of each layer, the filter/kernel size and the number of encoder, decoder blocks. As a rule, small and simple networks lack the complexity for classifying large image data as they are limited by the number of parameters. On the other hand, large and complex models can handle difficult classification tasks but come with a greater computational load due to their large number of parameters (Hay and Parthasarathy, 2018). For our task, the model architecture needed to be between these two limits. **Figure 6** shows an outline of the network architecture used. As can be seen, the network path is composed of 4 encoder blocks, 3 decoder blocks, and a final convolution block. Each encoder block consists of two consecutive convolution blocks followed by a max pooling layer. Each convolution block is composed of a convolution layer, followed by a Rectified Linear Unit (ReLU) (Nair and Hinton, 2010) layer and a batch normalization layer. Following the observations outlined in (Simonyan and Zisserman, 2014; Tran et al., 2015), we used small convolution kernels of size [3 × 3 × 3] in all convolution layers as they have been shown to improve computational efficiency and representation capability. All max pooling layers have a kernel size of [2 × 2 × 2] and a stride of [2 × 2 × 2] to effectively downsample inputs from the previous layer. Each decoder block is composed of an upsampling layer that reverses the process of max pooling performed at the encoder stage. This is followed by two consecutive convolution blocks, similar in structure to those used at the encoder stage. The last convolution block performs a single [1 × 1 × 1] convolution that reduces the number of output channels to the number of classes, so in our case, for a single marker, the final layer will reduce the output to a single channel. Finally, an additional sigmoid layer is used to normalize the network output so that the unnormalized logits can be fit to the target. During training an Adam optimizer is used with a starting learning rate of 0.0002 and a weight decay of 0.0001. Cropping is avoided in the decoder path by using padded convolutions, this ensures that the output resolution of the convolution layer is the same as that of the input. The resulting network architecture has a total of approximately 59 million learnable parameters.

In our case, upsampling via transposed convolution was favored over a pre-defined interpolation approach because it was observed that in cases of severe class imbalance, a learned upsampling approach performed better than simple interpolation. One possible reason for this could be that the input to the upsampling layer is rather coarse, and straightforward interpolation is too simple a process to capture subtle features. Due to this, small objects or regions (such as the marker being segmented here) are often overlooked or misclassified (Noh et al., 2015). This transposed convolution process reconstructs the previous spatial resolution and follows it with a convolution operation (Dumoulin and Visin, 2016).

The confocal volumes used here are of dimensions 1,024 × 512 ×32 in the *x*, *y*, and *z* directions, respectively. This is too large a volume of data to be input directly into the network as is, since at such sizes GPU memory becomes a limiting factor. This is a common problem when working with 3D data in deep learning methodologies. Instead of feeding in all data at once, we used an overlapping patch-based approach to train the network, with the full volume divided into patches of size 128 × 128 × 20. Patches are overlapped rather than tiled to ensure that the chance of a feature of interest (a nucleus in this case) occurring at the edge of a patch and being cropped is minimized. A 25% overlap between patches in all directions is used.

### Data Augmentation

We observed fairly consistent appearance between samples across time series and images, and so felt that extensive and complex augmentations such as elastic deformations, skewing and random angle rotations were not necessary during training. We first normalized the dataset samples to a zero mean and unit variance, followed by standard augmentation such as random flipping of the volume over the x-axis and random 90° rotations, representing different presentations of the root structure to the microscope. Given more complex datasets or perhaps different presentation to the microscope, additional augmentation of the data could be incorporated easily enough.

### Loss Function

Successful segmentation is made possible not only by the architecture of the network used, but also by the use of an effective loss function—a metric for the amount of error in a prediction—for the task at hand. The choice of loss function is even more important where there exists a severe class imbalance between the background and foreground objects. This is certainly the case here, where the fluorescent markers of interest represent a small proportion of the root, which itself occupies only a fraction of the complete image volume.

**Figure 7:**
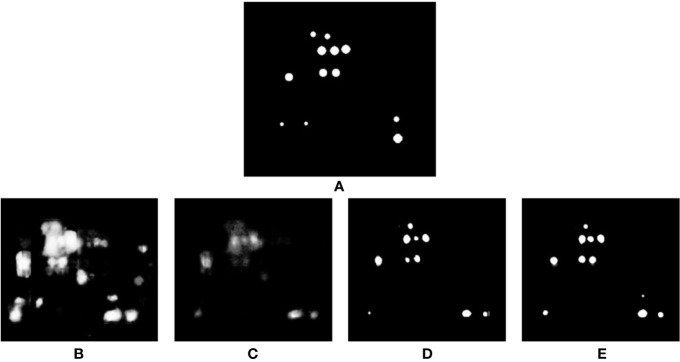
Comparison of segmentation results produced by using different loss functions. **(A)** shows the ground truth of the input volume labeled regions, network outputs are then shown using the following loss functions: **(B)** WCE (Weighted Cross Entropy), **(C)** BCE (Binary Cross Entropy), **(D)** Dice, and **(E)** GDL (Generalized Dice Loss).

Recently, in the literature, Weighted Cross Entropy (WCE) (Ronneberger et al., 2015), Binary Cross Entropy (BCE), Dice (Milletari et al., 2016), and Generalized Dice Loss (GDL) (Sudre et al., 2017) have been used as loss functions to segment data when there is a high imbalance between the foreground and background pixels. To find the best performing loss function for our dataset, separate instances of our network were trained using each of these loss functions, and the validation results produced by early stages of training were compared. **Figure 7** shows a comparison of the network output using different loss functions. As can be observed, for the binary segmentation of such an imbalanced dataset Dice and GDL appear to perform better than WCE and BCE. In the end, a decision was made in favor of GDL as it takes into consideration the class-rebalancing property of the Dice overlap (Sudre et al., 2017).

The GDL loss function was proposed by Crum et al. (Crum et al., 2006) to evaluate segmentation performance using a single score. Sudre et al. (Sudre et al., 2017) then proposed to use this score as a loss function to train deep neural networks to perform segmentation on highly imbalanced data and is represented as

(7) $$GDL=1-2\left[\frac{\sum_{l=1}^{L}w_{l}\sum_{n=1}^{N}t_{n}p_{n}}{\sum_{l=1}^{L}w_{l}\left(\sum_{n=1}^{N}\left(t_{n}+p_{n}\right)\right)}\right]$$

where *t* is the reference ground truth volume, *p* is the predicted probalistic map, and *w* is the label specific weight. The contribution of each label to the GDL weight *w* is determined by the inverse of that label’s volume. This therefore reduces the correlation between the Dice score and the region size. *w* is represented by

(8) $$w=\frac{1}{{\left(\sum_{n=1}^{N}t_{n}\right)}^{2}}$$

### Results

**Figure 8:**
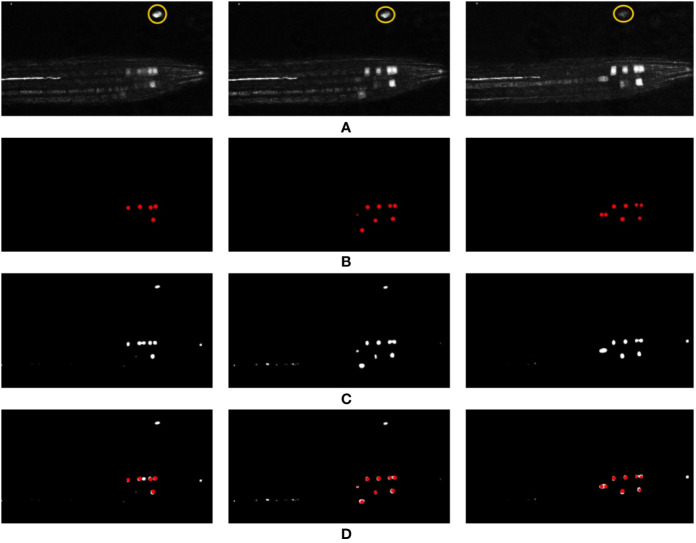
Representation of network output over different time points of the test timeseries (time points 1, 2, and 3), and comparison against the ground truth and the input data. **(A)** Represents a 2D slice from the input volume (note, the yellow circle indicates an artefact feature referred to in the main text), **(B)** represents the generated ground truth for this input volume, **(C)** represents the network output, and **(D)** shows the overlay of the ground truth (red) on top of the network output (white). If comparing to numerical results, note that this is a 2D section of a 3D slice—more nuclei are likely present.

In an effort to visualize the 3D output of our network for this paper, a 2D slice is taken from each volume along with its respective slice in the input volume and ground truth. This 2D slice representation has been used for the visualization of all results in **Figure 8**. Using this, the reader should be able to get a qualitative feel for network performance, but do note the input and output is a 3D volumetric image.

Despite limited training data, the challenge of working with the volumetric 3D data, and the artefacts and noise present in the confocal images, the results produced by the deep network are encouraging. This is especially true compared to the results of the classical machine learning studies presented earlier. The overlay of the ground truth over the output (see **Figure 8D**) shows that our network has detected almost all of the nuclei markers present in the input volumes along with some false positives; however, comparing these false positives with their respective input volumes shows that the network is arguably justified in detecting these features, as they are located on feasible artefacts in the image (represented as those enclosed within the yellow markers in **Figure 8A**). This clutter presents itself as a likely marker; however, its location in the image means a biologist would discount it. This is a notable error, and as such has been further discussed below.

In some places, the markers are in very close proximity. This is likely a common occurrence in this and other biological datasets, as the markers will tend to cluster at sites of similar biological action—in this case, cell division. It can be seen in, e.g., **Figure 8C** center panel that markers in close proximity are sometimes joined, producing a single output volume where there should be two distinguishable volumes in reality. Due to the shape of the regions, however, this error can be easily fixed in a post-processing stage using morphological operations, which will be explained in the discussion section.

As can be seen from **Figure 8**, the predicted results generated by the deep learning network are much closer to their respective ground truth volumes versus the earlier machine learning predictions. This is visible in the qualitative visual results, and also in the F-Measure result of **Table 3** versus the machine learning approaches in **Table 2**. Based on these encouraging results for deep learning, we take evaluation here a step further, and move to counting markers—that is, post-processing segmented pixels to locate and count the nuclear markers in the image. This would be the final desired result of our marker detection pipeline, which could be used to, for example, locate dividing cells in a time series, or provide a count of divisions in a time period. Here then, we move to an additional object/event-based analysis, rather than purely pixel-based. To do this, it is important to determine the criteria for what can be considered a “correct” detection and to distinguish between a true positive, false positive and false negative. This is accomplished by using a count and distance measure, where a simple Euclidean distance is determined between the center of a detected nucleus and its closest ground truth nucleus. If this distance is less than a set threshold, then it is considered as a true positive, otherwise it is considered as a false positive. Any nuclei that the network fails to detect are counted as false negatives. The value of this threshold was determined empirically and it was observed that a distance threshold of 5 pixels gave the best representation of the network’s performance. Furthermore, the area of all the objects detected by the network is determined and very small detections having a much smaller comparative area than the other detections are ignored, as these are clearly not nuclei. In practice, this value will be related to the size of the biological feature being marked, the zoom of the microscope, and the calibration scale of the image. **Table 3** reports the performance of each volumetric test sample given to the network using the deep learning model, providing both object count accuracies and the F-Measure of the pixel-based segmentation. In addition to producing more accurate results, the proposed deep learning method is a more practical solution in terms of inference time. On our local GPU, we experienced an inference time of approximately 10 min per input volume which is a huge improvement over the inference time of 12 h per input volume for Haralick features.

**Table 3:** Evaluation of proposed deep learning network using precision, recall, and f-measure for nuclei marker locations and per-pixel measurements, calculated over the six time points in the test time-series. For the sake of comparison, the per-pixel F-measure values written within brackets represent the best result achieved for this time stamp using machine learning.

Volumetric Time Stamp	Nuclei in Ground Truth	Nuclei in Predicted Output	True Positives	False Positives	False Negatives	Precision	Recall	F-Measure	Per-Pixel Measurements		
									Precision	Recall	F-Measure
**1**	5	8	5	3	0	0.63	1.0	0.77	0.65	0.26	0.37 (0.25)
**2**	11	9	8	1	2	0.88	0.8	0.84	0.84	0.21	0.34 (0.33)
**3**	9	9	8	1	0	0.88	1.0	0.94	0.84	0.25	0.39 (0.43)
**4**	6	6	5	1	0	0.83	1.0	0.90	0.77	0.35	0.49 (0.43)
**5**	3	6	3	3	0	0.50	1.0	0.66	0.61	0.45	0.52 (0.18)
**6**	2	5	2	3	0	0.40	1.0	0.57	0.46	0.42	0.44 (0.11)

### Discussion

It can be seen from the reported results that our network is producing encouraging results, even for difficult volumetric samples. Performance is particularly encouraging given the small number of training instances available. The network allows cell division markers to be identified, located and, if necessary, counted within an unseen volumetric dataset. The network continues to produce some false positives, however. Upon further investigation, we found that a false positive reported in almost every sample is actually an artefact (marked in **Figure 8A**) present in the test input volume throughout the entire time sequence. This artefact resembles a nucleus feature in appearance and is therefore reported as one by the network. **Figure 10** shows the presence of this artifact, which can be seen in close-up as the bright region at the top of the volume. Since this lies outside our pre-determined distance threshold to ground truth locations, it is reported as a false positive. Such an artefact is easy to throw away by visual inspection because of the context—the location is outside of the root, and so would be dismissed by a biologist. This highlights a downfall of our network; that context can be lost in the process of dividing the image into overlapping patches. It may be possible to build context into the network (indeed this is discussed in the Conclusion section), but here, we first test a simpler method to prevent detection by altering the training procedure.

**Figure 9:**
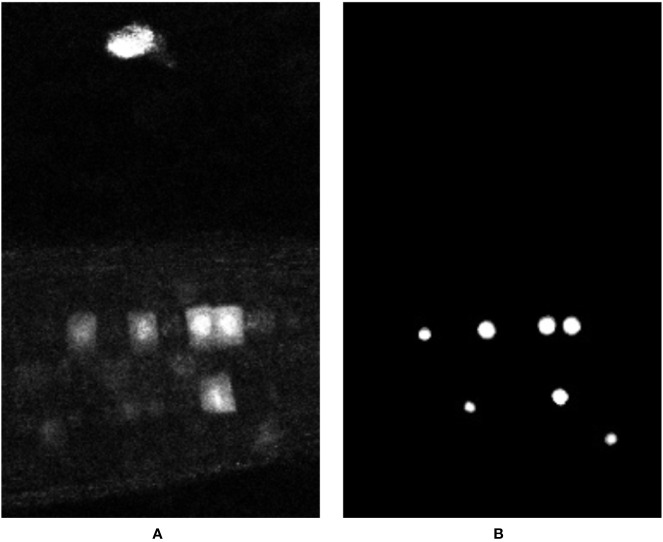
Representation of the output volume **(B)** for input data **(A)** produced by the network trained on the re-shuffled dataset, featuring a wider variety of training data. Note the artefact outside the root, at the top of the image, is no longer labelled on the output.

The underlying problem here is one inherent in machine learning—the training data did not include any such samples as negative ground truth. It is only present in the time-series that was randomly selected for testing the system. We hypothesize that had there been more training samples with such artefacts from which the network could learn, then the network could learn to ignore them. This underlines the importance of a having a representative training set when using machine learning. To address this, first, our network was re-trained from scratch on a smaller, re-shuffled dataset where the majority of the artefact stained samples were included into the training pool. But this caused a large imbalance of data as there were very few foreground samples compared to the background ones. To rectify this, the training/validation pool was reduced from 132 time points to around 50 time points, out of which 9 volumes had the artefact staining, and the remaining 41 did not. Two artefact stained time points were left out for the purpose of testing the network. As expected the system, successfully learned to ignore such artefacts and only reported and segmented the actual nuclei. The output of this re-trained network can be seen in **Figure 9**; here, the artefact is no longer reported even though it is very prominent in the input volume, demonstrating the importance of a suitable, representative training set.

**Figure 10:**
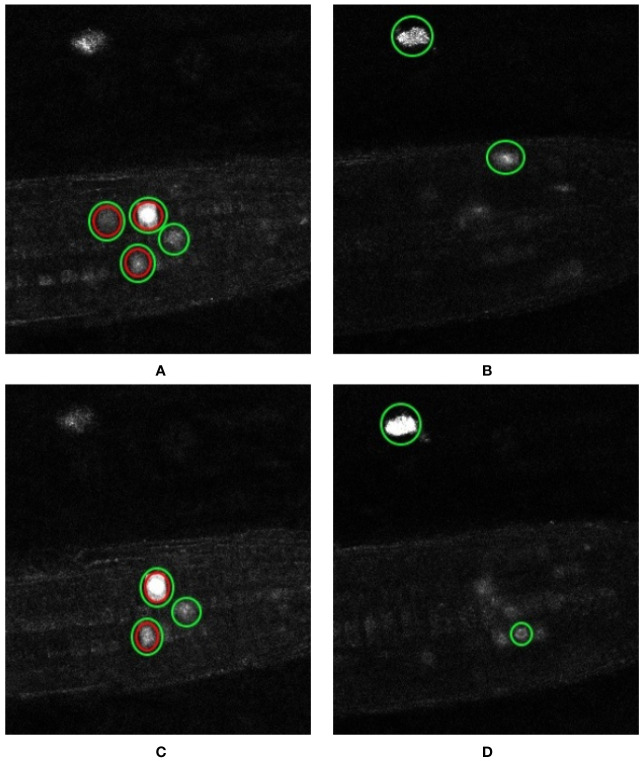
Error cases: artefact detection and ambiguous annotation. Representation of the nuclei detected by our network and those reported in the ground truth. Individual z-slices showing prominent detections taken from test volume 5 are shown in **(A**, **B)** while that from test volume 6 are shown in **(C**, **D)**. The nuclei enclosed in the green circles are the ones detected by the network and those enclosed in the red circles are the ones reported by the ground truth.

Another error case highlights the sensitivity of such deep learning approaches to annotations themselves. The network labels twice as many markers in volume 5 than are present in the ground truth (**Table 3**). **Figures 10A, B** show example z-slices from the input in question. Similarly, the network is reporting 5 nuclei present in volume 6, whereas only 2 are present according to the ground truth. As for test volume 6, **Figures 10C, D** show different z-slices from this volume where the nuclei have been detected. Green circles represent the network predictions, whereas red circles represent the ground truth locations. As can be seen, the network predictions seem reasonable (either partial marked locations or artefacts), but since they are not marked as present in the ground truth, these detections are reported as false positives. In the end, it would be left to the biologist to determine whether a detection by the network is a true positive or a false positive; it is likely a judgement call. This is clearly an issue with providing training data for—and a measure of confidence in the results of—similar deep network approaches; especially a challenge with large, 4D datasets as in use here.

**Figure 11:**
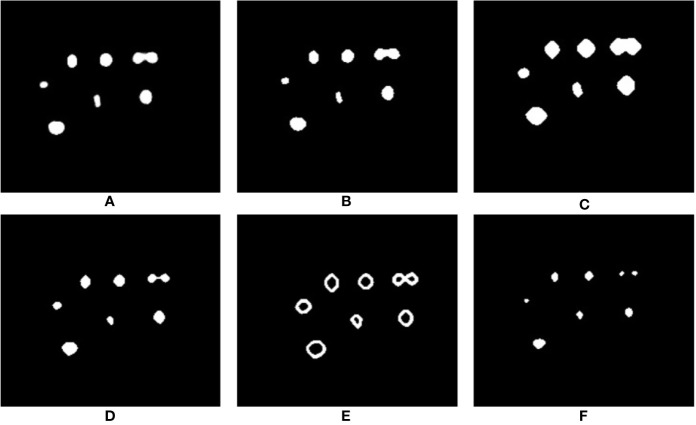
Steps in the post-processing process to distinguish incorrectly joined neighboring predictions. **(A)** The predicted output from the network; **(B)** morphological opening applied to remove unwanted noise; **(C)** morphological dilation marks the background pixels; **(D)** distance transform marks the foreground pixels; **(E)** subtraction of foreground from background to get the boundary region of connecting nuclei; **(F)** watershed segmentation produces the final result (images cropped to area of interest for clarity).

As shown in **Figure 8**, the network struggles to completely distinguish between nuclei that are closely located to each other, but is able to detect two entities that are joined (see **Figure 11A**). Despite the fact that point annotations are used, giving the regions of interest volumes means they can become connected regions as depicted in this figure. However, the shape they acquire when joined is amenable to further processing to distinguish the two regions. This allows us to make use of a combination of morphological operations to clean up the network output and further improve the result. **Figure 11A** shows a 2D slice taken from an output volume that has detected two nuclei as a single connected component; we will now use this volume to explain the post-processing process.

To begin with, the image quality of the output produced by the network is first improved by removing any unwanted noise and artefacts. This is achieved via morphological opening using a sphere structuring element. The output of this stage is shown in **Figure 11B**. Having cleaned up the volume, we may be confident that the region near the center of the connected components are the foreground and the region further away from the center are part of the background. The only region that we are unsure of is the boundary region that connects the two nuclei. To mark this boundary region we need to know which part of the connected components are background for certain, and which part of the connected components are foreground. The “sure background” region is obtained by morphological dilation of the volume using the same structuring element, the output of this step marks our “sure background” and is shown in **Figure 11C**. Distance transform is then applied on the dilated “sure background” volume, which is then thresholded to get the “sure foreground” region, as shown in **Figure 11D**. Now that we know for certain which areas represent background and which areas represent foreground we can identify the boundary region by subtracting the “sure foreground” volume from the “sure background” volume. The output of this subtraction is shown in **Figure 11E**. Having identified all the regions in the volume, the nuclei can be marked as areas of interest while the boundary regions can be marked as areas to ignore. Finally, watershed segmentation can make use of these marked areas to produce the final output that separates any connected nuclei at the boundaries, as can be seen in **Figure 11F**.

## Conclusion

We have presented segmentation approaches using classical machine learning, and a volumetric deep learning approach which is able to segment labeled nuclei markers in confocal time series datasets with only a limited number of training instances. Training has been carried out in time series datasets isolated from the test data, providing a realistic use case. The deep learning approach was found to perform with better accuracy than the machine learning algorithms, with less input required once the architecture of the networks has been determined. Features are learned rather than developed by hand, meaning given sufficient training data, performance is able to outperform traditional methods.

The segmentation here was carried out on discrete time points. A future development of this work will actively try to segment time-based events such as cell divisions, linking features over time perhaps using a tracking approach. This process will be performed on light-sheet data (versus confocal), as light-sheet microscopy is able to capture many more volumes both in frequency and period, but the work here will provide a foundation for this development.

The results demonstrated that unless particular care was taken with the training data coverage, it was easy for the network to mistake clutter for desired features, even if the clutter was located outside of the biological root system. The developed network makes use of volumetric patches in order to overcome memory demands on the GPUs. However, results here suggest contextual information could be useful to provide to the network, such that objects outside of biologically relevant areas could be discounted. Previous work has used lower resolution data with more spatial coverage to provide such context, and so such an approach could be utilized here in the future.

Of particular note is that the results were achieved with only a limited amount of simple augmentation. Given the relatively constrained problem we are handling - that of particular markers in a root oriented in the same direction in each image, captured using very similar microscope settings—these augmentations seem to be sufficient. However, there is potential work in developing confocal microscope specific augmentation. This could include, for example, synthesizing noise expected to affect confocal images, as well as simulating the effects of laser attenuation throughout the sample. Such augmentation could be expected to lessen the sensitivity of the training to particular features of the training set, which is even more important where training set size is limited—as is often the case with 3D microscopy due to the overhead in annotating the images, as well as the level of expertise required to do this.

In this paper, we have been concerned with finding fluorescent markers which indicate a growth event (in this case cell division) happening as part of the growth of a root. Therefore, when evaluating the approach, we are primarily interested in if we produce a prediction sufficiently close to a ground truth *x*, *y*, *z* label (a true positive) rather than a prediction away from one of these labels (a false positive). We also want to make sure ground truth labels are not missed (false negatives). In future work, we can refine the segmentation of the marker region itself, i.e., evaluate the pixel-wise segmentation of the region. While this resulting volume or shape information is less relevant for the marker in use here, it could be important if trying to analyze the shape of a cell as it expands or divides, for example. As a pixel-wise segmentation is already produced by the network, but refined in post-processing to a single location in space, the network is already partially capable of generating meaningful 3D shape labels.

## Data Availability Statement

The datasets and plugin used for this study can be found in the GitLab repository at https://gitlab.com/faraz.khan1/volumetric-segmentation-of-confocal-images.

## Author Contributions

FK designed and implemented the computational algorithms, models and experiments. MP wrote the annotation tool and provided guidance. UV performed biological experiments and annotation. AF managed the project and helped design the approaches, with MP and FK. All authors contributed to the article and approved the submitted version.

## Funding

This work was supported by the Biotechnology and Biological Sciences Research Council [grant number BB/N018575/1].

## Conflict of Interest

The authors declare that the research was conducted in the absence of any commercial or financial relationships that could be construed as a potential conflict of interest.
